# Frequency of Celiac Disease in Adult Patients with Typical or Atypical Malabsorption Symptoms in Isfahan, Iran

**DOI:** 10.1155/2012/106965

**Published:** 2012-04-01

**Authors:** Mohammad Hassan Emami, Soheila Kouhestani, Somayeh Karimi, Abdolmahdi Baghaei, Mohsen Janghorbani, Nahid Jamali, Ali Gholamrezaei

**Affiliations:** ^1^Department of Internal Medicine, Isfahan University of Medical Sciences, Isfahan, Iran; ^2^Poursina Hakim Research Institute, P.O. Box 81465-1798, Isfahan, Iran; ^3^Iranian Celiac Association, Isfahan, Iran; ^4^School of Public Health, Isfahan University of Medical Sciences, Isfahan, Iran

## Abstract

*Aim*. Atypical presentations of celiac disease (CD) have now been shown to be much more common than classical (typical) form. We evaluated the frequency of CD among adult patients with typical or atypical symptoms of CD. *Materials and Methods*. Patients referred to two outpatient gastroenterology clinics in Isfahan (IRAN) were categorized into those with typical or atypical symptoms of CD. IgA antitissue transglutaminase antibody was assessed and followed by duodenal biopsy. In patients for whom endoscopy was indicated (independent of the serology), duodenal biopsy was taken. Histopathological changes were assessed according to the Marsh classification. *Results*. During the study period, 151 and 173 patients with typical and atypical symptoms were evaluated (mean age = 32.8 ± 12.6 and 35.8 ± 14.8 years, 47.0% and 56.0% female, resp.). Frequency of CD in patients with typical and atypical symptoms was calculated, respectively, as 5.9% (9/151) and 1.25% (3/173) based on positive serology and pathology. The overall frequency was estimated as at least 9.2% (14/151) and 4.0% (7/173) when data of seronegative patients were also considered. *Conclusions*. CD is more frequent among patients with typical symptoms of malabsorption and these patients should undergo duodenal biopsy, irrespective of the serology. In patients with atypical symptoms, serological tests should be performed followed by endoscopic biopsy, and routine duodenal biopsy is recommended when endoscopic evaluation is indicated because of symptoms.

## 1. Introduction

Celiac Disease (CD), also known as gluten-sensitive enteropathy, is a genetic disorder affecting both children and adults. People with CD are unable to eat foods that contain gluten because, in these patients, gluten sets off an autoimmune reactions that cause the destruction of the small intestinal villi and leading to a malabsorption syndrome [1, 2]. While it was previously thought to be rare, epidemiological studies using sensitive and specific serological tests with biopsy verification established higher prevalence of CD (up to 1 : 100) in most countries [3–5].

Classical symptoms of CD in adults include chronic diarrhea, steatorrhea, and weight loss. Steatorrhea is associated with severe, extensive enteropathy, but it is often absent in patients whose disease is limited to the more proximal portions of the small intestine [2]. Classical symptoms of CD are present in less than 50% of the patients at presentation [1]. Abdominal discomfort and bloating are common at early presentation and often result in a mistaken diagnosis of more common gastrointestinal disorders such as irritable bowel syndrome (IBS) and dyspepsia for a long time, which contributes to a considerable delay in diagnosing CD [6, 7]. Untreated CD can be life threatening and increase the risk of certain types of cancer and lymphoma and also increase the risk of mortality compared to the general population [8]. Although there are no drugs to treat CD and there is no cure, a gluten-free diet (GFD) can lead to a normal and healthy live and can decrease the risk of malignancy and mortality [2, 8]. Therefore, prompt diagnosis of the disease and nutritional treatment is of great value. 

Despite the large epidemiological studies and screening and nutritional programs conducted in western countries, there are only a few investigations on the prevalence of CD in the general population in Asia, particularly Middle East [5, 9]. Epidemiological studies in Iran also are insufficient to provide an accurate estimation of CD among high risk or suspicious groups. Since there are few documented cases of CD in our society, it seems to be remained underdiagnosed [5].

There is still a controversy on cost-effectiveness and benefits of the population screening for CD [10, 11]. In the absence of a population screening program, targeting the screening to the certain high risk groups (case finding approach) can be an efficient use of the resources [10]. Epidemiological studies providing an estimated prevalence among different target groups (e.g., apparently healthy, suspicious CD, and high-risk groups) will enable us to establish further genetic, immunologic, and nutritional researches to control the disease. According to the wide aspects of presentation and variety of complications, and also regarding the lack of epidemiologic data on CD in Iran, we aimed to determine the prevalence of CD among patients referred with typical or atypical symptoms of malabsorption to see if routine screening of these patients is worthwhile.

## 2. Materials and Methods

### 2.1. Patients and Setting

This study was conducted between 2004 and 2005, on all patients with typical or atypical symptoms of CD referred to Poursina Hakim Research Institute (including two outpatient clinics of gastroenterology) in Isfahan, Iran. Classic or typical symptoms of CD were considered as chronic diarrhea, steatorrhea, and weigh loss. Atypical symptoms included unexplained abdominal pain, excessive gas passing, malodor stool or gas, constipation, intermittent diarrhea, bloating, and unexplained nausea or vomiting. The ethics committee of the Isfahan University of Medical Sciences approved the study and informed consent was obtained from all patients after explaining the aims and protocol.

### 2.2. Assessments

Data including demographics, clinical symptoms, complete past medical history, and associated disorders, and family history of CD were collected by a trained physician using a structured questionnaire. Laboratory data included thyroid function test, complete blood count (CBC), ESR, CRP, calcium, phosphor, and 3 times stool examination for all patients.

### 2.3. Serological Assessment for CD

The IgA antitissue transglutaminase (anti-tTG) antibody was measured for all patients using an enzyme-linked immunosorbant assay (ELIZA) technique by a commercially available kit (ORG540 A, ORGENTEC Diagnostica GmbH). The upper limit of the normal range (cut-off value) for t-TG IgA antibody, as determined by the manufacturer, was 10 u/mL. If the results was very low (<5 Au/mL), IgA level was measured to rule out IgA deficiency.

### 2.4. Pathological Assessment for CD

Endoscopic biopsy was recommended to all patients with typical symptoms, seropositive atypical cases, and those with IgA deficiency [1, 12]. Also, duodenal biopsy was done in seronegative patients with atypical symptoms, who had other indications of upper gastrointestinal endoscopy (due to their symptoms). Endoscopy was done with a standard 110 cm long video endoscope (EG 2940, Pentax EPM-3300), by a single gastroenterologist, during which at least four biopsy specimens were obtained from the distal part of the second portion of duodenum. The specimens were processed and stained with hematoxylin and eosin and studied under light microscopy by a gastrointestinal oriented pathologist. Histopathology was reported according to the modified Marsh classification [13]; Marsh type I: infiltrative phase with >30 lymphocytes per 100 enterocytes; Marsh II: infiltrative/hyperplastic phase; Marsh IIIA, IIIB, and IIIC: partial, subtotal, and total villous atrophy, respectively. Seropositive patients with at least Marsh I of villous atrophy and also seronegative cases with Marsh II or III of villous atrophy were considered to have CD if they had good response to GFD [12].

### 2.5. Statistical Analysis

The data were analyzed using SPSS software for Windows v 16.0 (SPSS Inc., Chicago, IL, USA). Comparisons were done with independent *t*-test or Mann-Whitney test for quantitative and Chi-square or Fisher's exact tests for qualitative data, and a *P* value of <0.05 was considered to be significant.

## 3. Results

During the study period, 151 patients with typical symptoms (mean age = 32.8 ± 12.6, 47.0% female) and 173 patients with atypical symptoms (mean age = 35.8 ± 14.8, 56.0% female) were evaluated. Comparisons of the two groups regarding demographic characteristics and symptoms are presented in Table 1. The differences between patients with typical and atypical symptoms in age and gender were not statistically significant (*P* > 0.05). The frequency of intermittent diarrhea and constipation was higher in atypical (*P* < 0.05) and fatigue/weakness in typical cases (*P* < 0.001). Duration of symptoms was longer in patients with atypical symptoms (*P* < 0.05).

### 3.1. Patients with Typical Symptoms for CD

In patients with typical symptoms, thyroid function test, stool exam, ESR and CBC results did not specify a diagnosis. Patients ≥ 50 years old (17 cases) underwent total colonoscopy and none of them had malignancy or inflammatory bowel disease. Family history of CD was not reported and IgA deficiency was not detected in any patient. Totally, 8.6% (13/151) of the patients with typical symptoms were seropositive for tTG-IgA, 12 patients accepted to undergo endoscopy. Histopathological studies showed Marsh IIIc in 4, Marsh IIIb in 3, and Marsh I in 2 patients. As upper gastrointestinal endoscopy was offered to all patients, 40.2% (56/139) accepted this procedure. Histopathological studies among these patients showed Marsh IIIc in 1, Marsh IIIb in 2, Marsh IIIa in 3, Marsh II in 1, and Marsh I in 9 patients. GFD was started in all patients with positive serology and a biopsy result suggestive of CD and in seronegative patients with Marsh III or II. One seronegative patient with Marsh IIIc and one with Marsh II did not respond to GFD and after more evaluation including colonoscopy, the patient with Marsh IIIc was diagnosed to have Crohn' disease. Other patients responded to GFD clinically and antibody became negative after six months in seropositive cases. Therefore, the prevalence of CD in patients with typical symptoms was calculated as 5.9% (9/151) based on positive serology and confirmed pathology and the overall prevalence was estimated as at least 9.2% (14/151) when data of seronegative patients were considered, as well. Patients' characteristics are shown in Table 2.

### 3.2. Patients with Atypical Symptoms for CD

In this group, 8 patients were diagnosed to have IgA deficiency, but none of them had CD. Totally, 2.8% (5/173) of the patients were seropositive for IgA anti-tTG, and in all of them duodenal biopsy was taken. Marsh II was shown in 3 of the patients, and 2 of them had normal histopathologic examination. As upper gastrointestinal endoscopy was offered to all patients with prolonged and unexplained symptoms, 37.5% (63/168) patients accepted this procedure. Among these patients, 2 had Marsh IIIa, 2 had Marsh II, and 6 Marsh I. GFD was started in all patients with positive serology and a biopsy result suggestive of CD and also in seronegative patients with Marsh III or II. All patients responded to GFD clinically and serology became negative in seropositive cases after six months. Finally, the prevalence of CD in patients with atypical symptoms was calculated as 1.25% (3/173) based on positive serology confirmed by pathology and the overall prevalence was estimated at least 4.0% (7/173) when data of seronegative patients were considered, as well. Patients' characteristics are shown in Table 2. The frequency of CD was higher in typical than in atypical patients (OR = 2.423, CI 95% = 0.95 to 6.17, *P* = 0.046 (one sided)).

Comparing patients with and without CD regarding presenting symptoms is presented in Table 3. Abdominal pain, diarrhea, bloating, and steatorrhea were more frequent in CD than non-CD patients (*P* < 0.05), but the differences regarding other symptoms were not statistically significant. Also, there was no significant difference between CD and non-CD cases in age or gender (*P* > 0.05).

## 4. Discussion

Previously, CD has been considered to be very rare in the Middle East and, based on this assumption, it was not generally considered as a possibility in the differential diagnosis of patients coming with nonspecific gastrointestinal symptoms [9]. By the development of more sensitive serological tests and a higher degree of disease suspicion, a marked increase in CD prevalence and incidence has been reported in recent decade [5]. Some evidence showed that a large proportion of patients present with atypical symptoms of malabsorption that can lead to misdiagnosis for a prolonged time [6, 14]. This delay can result in higher complications of CD such as different types of cancer and organ damage. Thus, prompt diagnosis of CD is of great important and while there is still a controversy on the cost-effectiveness of population screening for CD, case finding approach is more financially viable [10]. With this approach, we attempted to determine the frequency of CD in patients presenting with typical symptoms of malabsorption and those with nonspecific gastrointestinal complaints. We found that CD is present in about 9.2% of the patients coming with typical symptoms (12.1% (13/107) of the patients with chronic diarrhea) and 4.0% of those who come with atypical symptoms of malabsorption. Other studies from Iran also reported that CD is the most common cause of chronic nonbloody diarrhea in adults and children, ranging from 6.5% to 19% [15, 16]. These results indicate that classic presentation of malabsorption, specially with chronic diarrhea, is the main presentation of CD in our society. However, we found that 4.0% of patients coming to the outpatient clinics of gastroenterology with nonspecific symptoms of CD finally were diagnosed to have CD, which is much higher than that reported from screening studies of the general population in Iran, up to 1% [5]. Dyspepsia and IBS are the most common disorders diagnosed in outpatient clinics of gastroenterology. There are some studies with case finding approach that are done in these patients. The frequency of CD is reported from 1.4% [17] to 7% [18] in people with dyspeptic complaints and from 0.4% to 11.4% in patients with IBS [19–21]. A meta-analysis showed that biopsy-confirmed CD is 4-fold more prevalent in IBS patients than the general population (Pooled odds ratio = 4.34, CI 95% = 1.78–10.6) [22]. Studies with cost-effective analyses showed that testing for CD in patients with IBS-like symptoms is acceptable when the prevalence of CD is above 1% and it is a dominant strategy when the prevalence exceeds 8% and also in those with diarrhea predominant IBS [23, 24]. In spite of this evidence, most of the studies from Iran have shown no difference between patients with IBS-like symptoms and the general population in the frequency of CD [25]. More recent studies with large sample sizes also did not find a higher frequency of CD [20] even among patients with diarrhea-predominant IBS [19]. Therefore, decision for screening of these patients must be based on the population prevalence of CD, the accuracy of serological tests in that population, and the costs of IBS treatment [23].

An important finding in our study was the low sensitivity of serology (anti-tTG IgA antibody) in detecting CD patients specially in patients presenting with atypical symptoms. IgA anti-tTG antibody is the single most efficient serological test for the diagnosis of CD [26, 27]. It is well known that IgA anti-tTG levels correlate with the degree of intestinal damage, and that values can fluctuate in patients over time [28, 29]. Serological tests help in diagnosis of CD, but the gold standard is based on pathological study. We found that about 35% of CD patients with typical symptoms are seronegative that shows the best method for diagnosing CD in these patients is a panel of serological tests and endoscopic biopsy together, which is previously recommended by other investigators [1]. In patients with non-specific gastrointestinal symptoms we found positive serology in less than half of the patients. Accordingly we recommend that diagnostic approach in such patients should be started with serological tests and when the endoscopy is indicated for evaluation of the symptoms, duodenal biopsy and evaluation for CD histopathology should be considered. During the endoscopy, the presence of features of villous atrophy (such as scalloping of mucosal folds, absent or reduced duodenal folds, or a mosaic pattern of the mucosa) has a high negative predictive value for CD and could be helpful in decision for biopsy [30, 31]. However, such features have a very low sensitivity [31, 32] and also evidence has shown that duodenal biopsies reveal other abnormalities and could be helpful in patients with chronic diarrhea and/or abdominal pain for further following workups [33]. Therefore, we recommend routine duodenal biopsy in endoscopic evaluation of patients referring with nonspecific gastrointestinal symptoms.

## 5. Conclusion

We found that CD is more prevalent among patients referring with typical symptoms of malabsorption specially chronic diarrhea than patients with atypical symptoms. Any patient who has classic symptoms of CD should undergo duodenal biopsy, irrespective of whether serologic testing for CD has been performed or was positive. In patients with atypical symptoms, serological tests should be performed followed by endoscopic biopsy. In these patients, routine duodenal biopsy is recommended when endoscopic evaluation is indicated.

## Figures and Tables

**Table 1:** Patients' characteristics.

	Typical	Atypical	**P**
	*N* = 151	*N* = 173	
Age (year)	32.8 ± 12.6	35.8 ± 14.8	0.057*
Male/Female	80 (52.9%)/71 (47.0%)	76 (43.9%)/97 (56.0%)	0.065**
*Symptoms*			
Chronic Diarrhea	107 (70.8%)	—	—
Steatorrhea	40 (26.4%)	—	—
Weight Loss	64 (42.3%)	—	—
Abdominal Pain	80 (52.9%)	79 (45.6%)	0.115**
Bloating	68 (45.0%)	75 (43.3%)	0.424**
Intermittent Diarrhea	8 (5.2%)	26 (15.0%)	0.005**
Constipation	30 (19.8%)	60 (34.6%)	0.002**
Flatulence	65 (43.0%)	88 (51.4%)	0.098**
Fatigue/Weakness	56 (37.0%)	30 (17.3%)	<0.001**
Symptom Duration (Month)	36.3 (SE = 3.6)	54.7 (SE = 5.7)	0.008*

Data are presented as mean ± SD (SE) or number (%).

*Independent Sample *t*-Test or Mann-Whitney Test.

**Chi-Square or Fisher's Exact Tests.

**Table 2:** Patients with CD and typical/atypical symptoms.

	Typical	Atypical	**P**
	*N* = 14	*N* = 7	
Age, year	33.5 ± 13.0	39.2 ± 12.3	0.795*
Male/Female	6 (42.8%)/8 (57.1%)	3 (42.8%)/4 (57.1%)	0.676**
Positive serology	9 (64.2%)	3 (42.8%)	0.319**
*Marsh classification*			
I	2 (14.2%)	0	0.004**
II	0	5 (71.4%)	
IIIA	3 (21.4%)	2 (28.5%)	
IIIB	5 (35.7%)	0	
IIIC	4 (28.5%)	0	

Data are presented as mean ± SD (SE) or number (%).

*Independent Sample *t*-test or Mann-Whitney test.

**Chi-Square or Fisher's Exact Tests.

**Table 3:** Comparison of symptoms between patients with and without CD.

	CD	Non-CD	**P**
	*N* = 21	*N* = 303	
Age (year)	32.8 ± 12.6	35.8 ± 14.8	0.057*
Male/Female	9 (42.8%)/12 (57.1%)	147 (48.5%)/156 (51.4%)	0.065**
Abdominal Pain	15 (71.4%)	144 (47.5%)	0.028**
Diarrhea	13 (61.9%)	120 (39.6%)	0.039**
Bloating	15 (71.4%)	128 (42.2%)	0.009**
Constipation	3 (14.2%)	87 (28.7%)	0.117**
Weight Loss	5 (23.8%)	59 (19.4%)	0.402**
Flatulence	9 (42.8%)	144 (47.5%)	0.427**
Fatigue/Weakness	9 (42.8%)	77 (25.4%)	0.072**
Steatorrhea	6 (28.5%)	34 (11.2%)	0.032**

Data are presented as mean ± SD (SE) or number (%).

*Independent Sample *t*-test or Mann-Whitney test.

**Chi-Square or Fisher's Exact Tests.
